# Application of Machine Learning for the in-Field Correction of a PM_2.5_ Low-Cost Sensor Network

**DOI:** 10.3390/s20175002

**Published:** 2020-09-03

**Authors:** Wen-Cheng Vincent Wang, Shih-Chun Candice Lung, Chun-Hu Liu

**Affiliations:** 1 Research Center for Environmental Changes, Academia Sinica, Nangang, Taipei 115, Taiwan; phdzen@gate.sinica.edu.tw (W.-C.V.W.); lch0909@gate.sinica.edu.tw (C.-H.L.); 2 Department of Atmospheric Sciences, National Taiwan University, Taipei 106, Taiwan; 3 Institute of Environmental Health, National Taiwan University, Taipei 106, Taiwan

**Keywords:** efficient in-field PM_2.5_ correction, random forest model, particle sensing correction, in-field calibration, PM sensing device

## Abstract

Many low-cost sensors (LCSs) are distributed for air monitoring without any rigorous calibrations. This work applies machine learning with PM_2.5_ from Taiwan monitoring stations to conduct in-field corrections on a network of 39 PM_2.5_ LCSs from July 2017 to December 2018. Three candidate models were evaluated: Multiple linear regression (MLR), support vector regression (SVR), and random forest regression (RFR). The model-corrected PM_2.5_ levels were compared with those of GRIMM-calibrated PM_2.5_. RFR was superior to MLR and SVR in its correction accuracy and computing efficiency. Compared to SVR, the root mean square errors (RMSEs) of RFR were 35% and 85% lower for the training and validation sets, respectively, and the computational speed was 35 times faster. An RFR with 300 decision trees was chosen as the optimal setting considering both the correction performance and the modeling time. An RFR with a nighttime pattern was established as the optimal correction model, and the RMSEs were 5.9 ± 2.0 μg/m^3^, reduced from 18.4 ± 6.5 μg/m^3^ before correction. This is the first work to correct LCSs at locations without monitoring stations, validated using laboratory-calibrated data. Similar models could be established in other countries to greatly enhance the usefulness of their PM_2.5_ sensor networks.

## 1. Introduction

Millions of premature deaths worldwide can be attributed to particulate matter with an aerodynamic diameter less than or equal to 2.5 μm (PM_2.5_) [1,2], which is one of the human carcinogens classified by the International Agency for Research on Cancer [3]. Rising PM_2.5_ levels in the ambient air and their associated health impacts are important environmental health issues that concern the general public, especially in developing countries [4,5]. In eastern Asia during 1998–2000, 51% of the population lived in areas with annual mean PM_2.5_ levels above the recommended guideline of the World Health Organization (35 μg/m^3^). This percentage increased to 70% during 2010–2012 [6], showing the deterioration of the air quality in this region.

In resource-limited Asian countries, there are insufficient numbers of regulatory monitoring stations in urban areas with high population densities. The purpose of the PM_2.5_ monitoring stations of Environmental Protection Administrations (EPAs) worldwide is to assess the well-mixed ambient pollutant levels. Therefore, such monitors are situated at a height of 10–15 m above the ground. However, the intensive emissions of community pollution sources in Asia, such as restaurants and temples, result in high PM_2.5_ levels in the immediate living environments of citizens at street level [7,8,9,10]. Even living in the same airshed, residents from different communities with different emission sources are exposed to different PM_2.5_ levels, demonstrating the need for community air monitoring. In Taiwan island, there are 57 regular air monitoring stations, covering an area of 35,887 km^2^ [11]. Excluding the two-thirds of mountainous areas with little population, on average, every 300 km^2^ of land area has only one air quality station. Even in the capital area (Taipei metropolitan), every 141 km^2^ has only one air quality station on average. Thus, there are still large monitoring gaps between these stations. These gaps can be filled by the observations provided by low-cost sensors (LCSs) for ambient-level and street-level air quality monitoring.

The United Stations Environmental Protection Agency (USEPA) noted that LCSs can be used to fill the observational gaps of environmental monitoring after appropriate quality assurance and quality control procedures [12]. Currently, there are more than 4000 PM_2.5_ LCSs in the citizen air quality network (CAQN [13]) in Taiwan as a result of the collaboration of citizens, private enterprises, and non-governmental organizations (NGOs) with partial government funding. The PM_2.5_ sensor network on the Island of Taiwan is possibly one of the most densely distributed sensor networks in the world [14,15], with (on average) three LCSs per 1 km^2^. CAQN has increased the environmental awareness of Taiwanese citizens dramatically. However, these sensors installed by citizens or NGOs are not calibrated, and data accuracy is not assured. Thus, their readings may be 2–3 times the actual concentrations [10]. Such limitations in data quality have restrained the application of these sensors in research and policy development.

LCSs need to be calibrated, since their readings usually deviate from those of research-grade instruments [10]. The actual cut-off points of particle sizes for LCSs are not consistent with the statements declared by the manufactures [16]; individual correction equations are, thus, needed for each sensor, even those from the same batch [10]. Most environmental scientists evaluate the performance of LCSs with side-by-side comparisons against research-grade instruments in the laboratory or in the field. Research-grade instruments, which are considered the golden standard for such comparisons, include instruments with measuring principles based on light scattering, oscillating tapered elements, β-attenuation, and filter-weighing, such as GRIMM instruments [10,17,18,19,20], TSI SidePake AM510 [21,22], tapered element oscillating microbalance [18,23], β-attenuation monitor [17,18,19,24,25], and ultrasonic personal aerosol sampler [26]. Correction equations are established to adjust the LCS readings to those of research-grade measurements. Mathematical models, such as linear regression [17,18,19,20,27] or multiple linear regression (MLR) models [23,25,28], have been used to establish these correction equations. The latter models usually consider ambient environmental factors.

To obtain accurate PM_2.5_ levels for LCSs, data correction is a necessary procedure. For thousands of PM_2.5_ sensors already installed by citizens in the field, an innovative method for correction is needed, since traditional laboratory or field evaluations with side-by-side comparisons require a great deal of human resources and time. Furthermore, it is impractical to correct thousands of sensors. Innovative data science techniques, such as machine learning, could also be used for correcting LCS readings [29]. Machine learning techniques have been used for air pollution issues for different purposes. Cheng et al. applied transfer learning to correct the PM_2.5_ values of the citizen network in Beijing, China using the PM_2.5_ levels from public environmental monitoring stations [30]. Pandey et al. predicted the levels of ultrafine particles in China through a machine learning approach [31]. Hsieh et al. used an artificial neural network and deep learning to analyze the proper position to install air pollution stations in Beijing [32]. Zheng et al. analyzed and predicted the air quality in different areas through data mining and machine learning using data from 43 cities in China [33]. Paas et al. used an artificial neural network to acquire the mass concentrations and numbers of different sizes of particles in Germany [34]. Peng et al. forecasted the levels of ozone, PM_2.5_, and NO_2_ in Canada using multiple linear regression and multi-layer perceptron neural networks [35]. Moreover, machine learning methods may provide a significant accuracy improvement for gaseous and particle sensors [23,36].

Therefore, we proposed a hybrid method combining laboratory evaluations and data science to ensure that the LCS networks provide accurate PM data [20]. First, the LCS data are corrected through side-by-side laboratory comparisons for “seed” LCS devices, which can be installed strategically in areas without EPA stations; secondly, statistical or machine learning methods are applied to adjust nearby uncalibrated LCS devices using data from the EPA stations or the seed LCS devices wherever available. In this way, readings from uncalibrated LCS devices in the CAQN can be corrected to nearly research-grade observations. The first part of obtaining reliable and robust correction equations is to convert the readings of LCS devices to research-grade (or FEM-comparable) measurements via side-by-side comparisons with research-grade instruments in the laboratory, as presented in [20].

The current work focuses on the second part of this process: Applying machine learning to correct data from the LCS network with the PM_2.5_ values from Taiwan EPA stations. The objectives of this work are: (1) To establish data correction models based on machine learning techniques with the PM_2.5_ data from the Taiwan EPA to correct the readings of the sensor network; (2) to evaluate the model performance with data from the same sensor network calibrated with laboratory evaluations; and (3) to explore the best data correction models using choices of computing efficiency and day/night periods. As can be seen in the later section, using in-field corrections with machine learning techniques, the PM_2.5_ data quality of LCSs can be greatly improved. Furthermore, this is the first work to introduce the use of a nighttime dataset instead of a whole-day dataset for the establishment of a data correction model with machine learning in order to prevent interference from local emissions during the daytime. This method can be further used to conduct in-field corrections for CAQN in Taiwan, as well as for other sensor networks in other countries.

## 2. Materials and Methods

### 2.1. Sensor Network Introduction

The LCS network corrected by Taiwan EPA data in this work consists of LCS devices designed for research purposes, namely, AS-LUNG-O. LCS devices integrate LCS, power, and data transmission components. AS-LUNG-O is an LCS device integrated by our team and designed for long-term outdoor monitoring for scientific research [10]. AS stands for Academia Sinica (the research institute that supports its development), while LUNG indicates the human organ most commonly affected by air pollutants, and O indicates the “outdoor” version. AS-LUNG-O (~650 USD basic manufacturing costs) incorporates sensors for PM_2.5_ (PMS3003, Plantower, Beijing, China), CO_2_ (S8, Senseair AB, Sweden), temperature/humidity (SHT31, SENSIRION, Staefa ZH, Switzerland), and Global Positioning System (GPS, u-blox, Switzerland). The PM_2.5_ sensor, PMS3003, has been evaluated by several research teams in laboratory environments. For example, Kelly et al. [18] obtained an R^2^ of 0.73–0.97 in wind tunnels, and Sayahi et al. [37] obtained an R^2^ > 0.978 for 242 sets of PMS3003 in a controlled chamber. These results indicated the good performance of PMS3003 compared to research-grade instruments.

The sensors for AS-LUNG-O are placed in a waterproof shelter connected to a solar panel with backup batteries for the power supply, with the option to use household electricity, where easily accessible. The size of the whole set is roughly 60 cm (W) × 50 cm (D) × 50 cm (H), with a weight of approximately 4.8 kg. Data can be transmitted wirelessly using the built-in 4G modules to a cloud database with one-min intervals. An SD card was added as a complement to avoid data loss during wireless transmission. Currently, most of the LCSs used in the CAQN (http://www.aqmd.gov/aq-spec/product/edimax) in Taiwan are PMS5003 (Plantower, Beijing, China), which is also a Plantower LCS.

AS-LUNG-O is a versatile LCS device capable of operating under the harsh weather conditions in subtropical Taiwan, which experiences high humidity (e.g., The mean relative humidity (RH) was 74% in the year of 2016 [10]) and frequent Typhoons [38]. AS-LUNG-O can be installed on the light poles in the streets and was used in a small town in a mountainous area to fill the data gaps of PM_2.5_ monitoring. The incremental PM_2.5_ concentration increases due to different community sources were, thus, quantified using AS-LUNG-O [10]. Therefore, other communities without EPA monitoring stations can also use AS-LUNG-O to acquire the PM_2.5_ levels.

**Figure 1:**
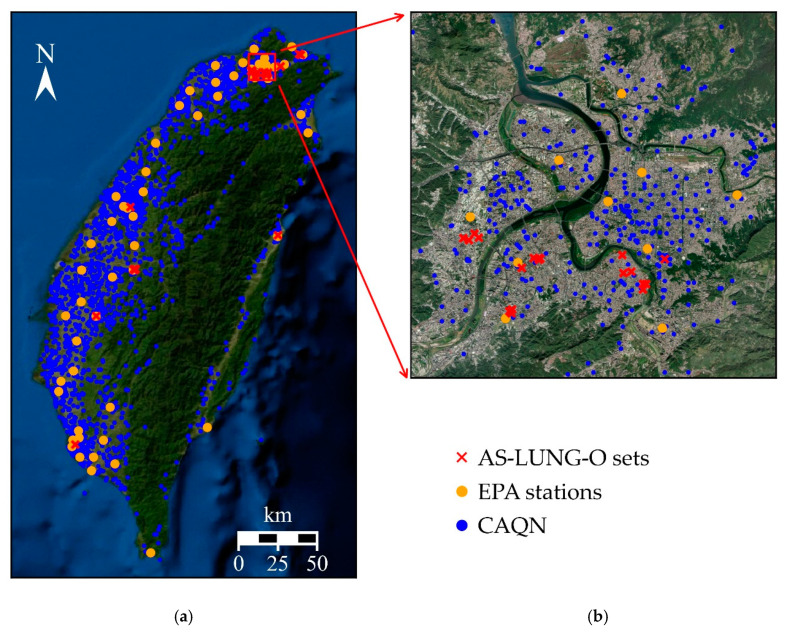

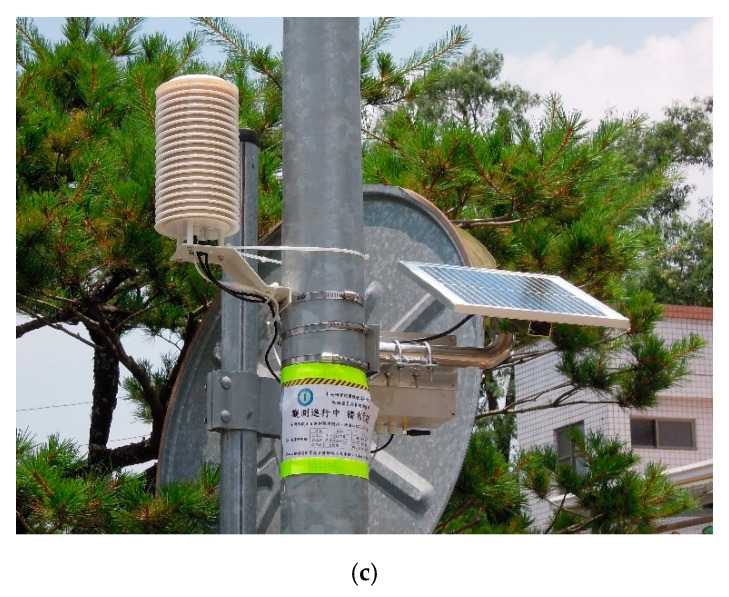
Distribution of AS-LUNG-O sets, Environmental Protection Administration (EPA) stations, and sensors of the citizen air quality network (CAQN) in (**a**) the whole of Taiwan island, (**b**) the Taipei metropolitan area from July 2017 to December 2018, and (**c**) an AS-LUNG-O set at street level.

This work uses data from the AS-LUNG-O network, including 39 AS-LUNG-O sets installed in different communities since July 2017 around Taiwan (Figure 1). Twenty-eight sets were installed in urban communities in Taipei’s metropolitan areas with high population densities in northern Taiwan, while two sets were placed in the suburban communities of Taipei. In addition, 6, 2, and 1 set(s) were installed in central, southern, and eastern Taiwan, respectively. Out of the 28 sets in the urban communities in Taipei, 27 were installed near certain community sources such as traffic, restaurants, temples, night markets, etc., as described in [10]. These 27 sets were set-up at street-level on light poles around 2–2.5 m above the ground. The other 12 sets were set-up at a high-level (around 10–15 m above the ground) on the rooftops of elementary schools or government buildings to assess the PM_2.5_ in ambient air.

The AS-LUNG-O network is considered a research-grade sensor network, since the data of AS-LUNG-O were corrected using correction equations based on laboratory evaluations with side-by-side comparisons against a research-grade instrument for every AS-LUNG-O reading [10]. The research-grade instrument used in these laboratory evaluations was GRIMM 1.109 (GRIMM Aerosol Technik GmbH and Co. KG, Ainring, Germany). The data from GRIMM 1.109 were in excellent agreement (R^2^ = 0.999, with a bias of roughly ±11%) with the data from an EDM-180 (GRIMM Aerosol Technik Ainring GmbH and Co, Ainring, German) [20], an FEM instrument designated by the USEPA for PM_2.5_. The mean values of R^2^ for the correction equations were 0.97, with ranges from 0.82 to 0.99 for these 39 sets. Without data correction, AS-LUNG-O overestimates PM_2.5_ by about 1.5–2.9 times [10,20]. PM_2.5_ observations with 1 min resolutions from AS-LUNG-O were converted to GRIMM comparable measurements according to the correction equations and saved in the cloud database for this AS-LUNG-O network.

This research-grade AS-LUNG-O network provides a great opportunity to evaluate the feasibility and performance of the correction models based on machine learning techniques. The raw PM_2.5_ readings of AS-LUNG-O corrected by the laboratory correction equations are “GRIMM-calibrated PM_2.5_”, while those corrected by the machine learning techniques are “model-corrected PM_2.5_”. The performance of the machine learning correction models can be evaluated by comparing the GRIMM-calibrated PM_2.5_ with model-corrected PM_2.5_.

### 2.2. The Data Correction Models

This work applied machine learning techniques to correct the raw readings of AS-LUNG-O sets with those of Taiwan EPA monitoring stations. Only EPA stations within 3 km of the AS-LUNG-O sets were selected in our work. Out of the 57 regular Taiwan EPA stations, 11 stations were selected, including 6 stations in the north, 2 in central Taiwan, 2 in the south, and 1 in the east. Hourly measurements of 24 h during July 2017 and December 2018 from these stations were used to establish the data correction models. The instruments used in Taiwan EPA [39] are Met One BAM-1020 (Met One, Inc., Grants Pass, OR, USA) and VEREWA-F701 (VEREWA, Ltd., Germany).

To establish in-field data correction models, two machine learning techniques (introduced below) were used in this work and compared with a correction model established using traditional MLR. These three models used PM_2.5_ data from EPA stations as their simulation targets to adjust the raw readings of AS-LUNG-O sets within a 3 km radius. These models were constructed using a personal computer environment with an Intel^®^ CoreTM i7-8700 and 32 GB RAM.

The inputs for these models were: (a) The raw PM_2.5_ readings, (b) the temperature, RH, latitude, and longitude of the AS-LUNG-O sets, (c) the PM_2.5_ levels of the nearest EPA station, and (d) the distance between AS-LUNG-O and the EPA station. These inputs (from July 2017 to December 2018) were used for 10-fold cross-validation (90% of data randomly selected for the training set; the others for the validation set) and holdout validation (50% of data randomly selected for the training set; the others for the validation set) tests to evaluate the robustness of these three models. Since there were only 1.5 years of data, we used the holdout method to generate the correction models; therefore, most of the data could be kept to validate the models, which can avoid to overvaluing the performance of the models under the situation of only using less data (10% of data) for the evaluation. Leaving more data in the validation set (50% of data in the holdout validation in this study) could increase the power of the model estimation. [40,41]. Data correction models were constructed using the training dataset, and the validation dataset was used to evaluate the correction accuracy of the models built. The MLR and machine learning models are introduced below.

The MLR model is established as follows:

(1) $${PM}_{2.5_{target}}=\beta_{0}+\beta_{1}\times {LCS}_{{PM}_{2.5_{raw}}}+\beta_{2}\times T+\beta_{3}\times RH+\beta_{4}\times Month+\beta_{5}\times Day\;\;+\beta_{6}\times Hr+\beta_{7}\times lat+\beta_{8}\times lon+\beta_{9}\times D$$

where $\beta_{0}$ is the intercept; $\beta_{1}–\beta_{9}$ are the regression coefficients; ${PM}_{2.5_{target}}$ is the simulation target of the correction model, EPA PM_2.5_; ${LCS}_{{PM}_{2.5_{raw}}}$ is the raw readings from AS-LUNG-O (μg/m^3^); T is the temperature (°C); RH is relative humidity (%); Month, Day, and Hr (hour) are the time values of the observations; lat and lon are the latitude and longitude of the AS-LUNG-O sets; and D is the distance of the AS-LUNG-O and the nearest EPA station (km).

Two machine learning techniques used in this work were support vector machine (SVM) and random forest (RF). SVM is based on the generalized portrait algorithm developed in the 1960s by Russian mathematicians and is a supervised learning algorithm used for classification [42,43]. The SVM algorithm is a popular machine learning tool that offers solutions for both classification and regression problems. The objective of SVM is to build an optimal hyperplane as a classifier in high dimensional space, and the data points closest to the hyperplane are called support vectors. New data are then divided by that classifier and predicted to belong to a category based on the hyperplane [44]. Our present work applies SVM to construct the support vector regression (SVR) model.

The random forest model is an ensemble learning method for classification and regression that builds a multitude of decision trees during the training process and constructs the modes of the classes or the mean predictions for classification and regression [45,46,47]. Using the random subspace method to build decision trees was first proposed by Ho et al. [45]. Breiman [46] further proposed to use the bagging algorithm to generate random forest to avoid over-fitting in the decision trees. The learning targets are numerical variables rather than class labels [46,47]. Our present work applies a random forest to construct a random forest regression (RFR) model.

### 2.3. Evaluation of the Correction Models

**Figure 2:**
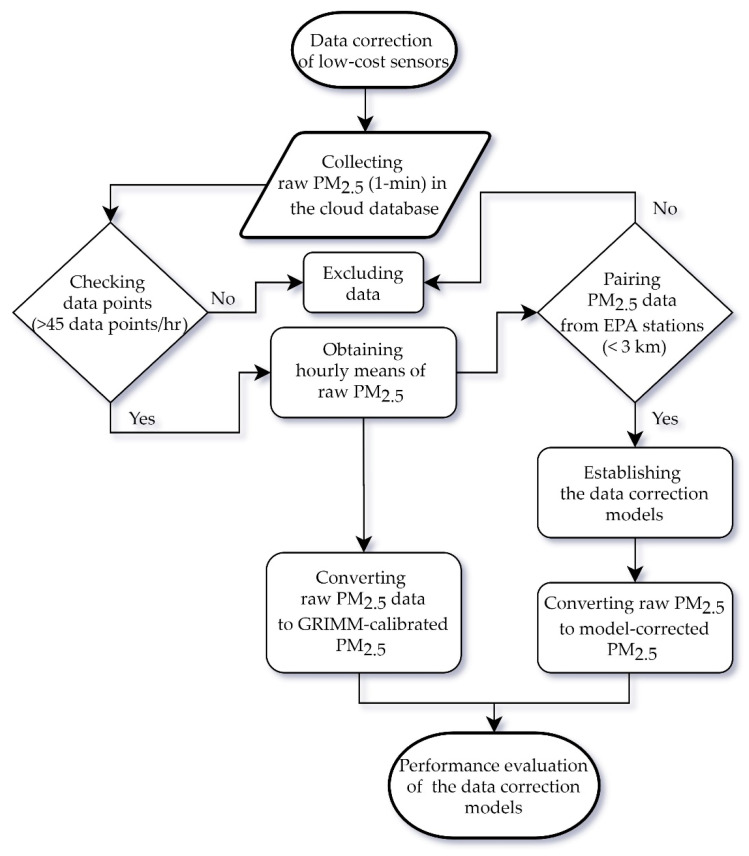
Flow chart of the data correction process.

Figure 2 shows a flow chart of the data correction process. Raw PM_2.5_ readings with a 1 min resolution were averaged to their hourly means to match the hourly observations from the nearest EPA stations within a 3 km radius. If the numbers for the raw PM_2.5_ in one hour were less than 45, this hourly mean was discarded. After collecting all aforementioned input data, data correction models with three different methods can be established. A model-corrected PM_2.5_ based on the optimal correction model can be obtained and then compared with the GRIMM-calibrated PM_2.5_ corrected based on traditional laboratory evaluations. In this way, the performance of the PM_2.5_ correction model can be evaluated accordingly.

Since the differences between the AS-LUNG-O readings and EPA observations may be affected by the community sources (of which emission activities change over time), the correction model with the best performance will be constructed based on whole-day (24 h) or nighttime (00:00–06:00) periods. The latter period was chosen because most of the community PM_2.5_ sources associated with human activities were minimal during this period. The optimal correction model built with data from nighttime patterns can be used to obtain the systematic relationships of data from AS-LUNG-O and EPA instruments without interference from nearby sources around the locations of the AS-LUNG-O sets.

The indicators used for evaluating model performance are root mean square error (RMSE), Pearson correlation coefficient (r), and coefficient of determination (R^2^). R^2^ is used to assess the predictive or explanatory ability of the model and should be close to 1, while r shows the correlations between two variables. The equation of RMSE is as follows:

(2) $$\mathrm{RMSE}=\sqrt{\frac{\sum_{i=1}^{n}{\left(Y_{i}-M_{i}\right)}^{2}}{n}}$$

The values of RMSE represent the difference between the model-corrected PM_2.5_ (Mi) and referenced PM_2.5_ levels (Yi) (EPA PM_2.5_ used in the selection evaluation for the machine learning methods; GRIMM-corrected PM_2.5_ used in the performance evaluation of the selected correction model). Thus, the closer these values are to zero, the better the model performs. Additionally, for the final model, mean absolute errors (MAEs) were also calculated for comparison with those from literature.

## 3. Results

### 3.1. Measurements of AS-LUNG-O Sets and EPA Stations

Table 1 shows a summary of the raw PM_2.5_ of the AS-LUNG-O sets and the PM_2.5_ observations of the nearest EPA stations during July 2017 and December 2018, as classified by different seasons. The range of PM_2.5_ for the EPA stations during this period is 2.0–135.0 µg/m^3^. It can be seen that the highest PM_2.5_ means and the maximum PM_2.5_ occurred during winter for both the AS-LUNG-O and EPA PM_2.5_ levels. The raw PM_2.5_ values of the AS-LUNG-O sets were, on average, higher than those from the EPA by about 1.9–2.2 fold. These data were used to establish and evaluate the three data correction models.

### 3.2. Performance Evaluation of the Correction Models

**Figure 3:**
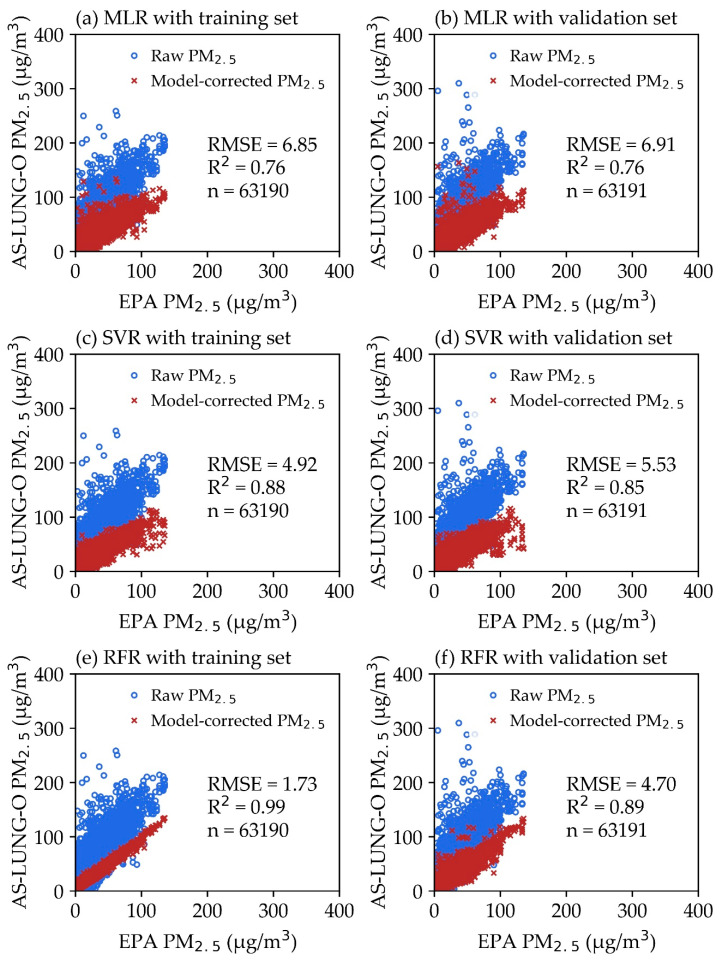
Model performance of (**a**) multiple linear regression (MLR) with a training set, (**b**) MLR with a validation set, (**c**) support vector regression (SVR) with a training set, (**d**) SVR with a validation set, (**e**) random forest regression (RFR) with a training set, and (**f**) RFR with a validation set. The RMSE, R^2^, and n are listed in the graphs.

We conducted 10-fold cross-validation and holdout validation tests to evaluate the robustness of the models. The results of the 10-fold cross-validation test for MLR, SVR, and RFR were based on the same training and validation datasets. The average values of RMSE and R^2^ for the results of the 10-fold cross-validation test were 6.88 ± 0.10 μg/m^3^ and 0.76, 5.23 ± 0.08 μg/m^3^ and 0.86, and 4.36 ± 0.06 μg/m^3^ and 0.91, for MLR, SVR, and RFR, respectively. The results of the holdout evaluation test were presented in Figure 3. The differences between the RMSEs of the 10-fold cross-validation and the holdout validation tests were about averagely 0.03, 0.30, and 0.34 μg/m^3^ for MLR, SVR, and RFR, respectively. Figure 3a–f show the distribution of model-corrected PM_2.5_ from the AS-LUNG-O sets and EPA PM_2.5_ data in the training and validation sets with three different data correction models. Based on the same training set of 63,190 data points, the computation time is 0.01, 8.16, and 0.23 minutes for building models MLR, SVR, and RFR, respectively. The R^2^ of 0.76–0.99 for these three models shows these models have good explanatory abilities. In terms of RMSE, RFR is the best model (1.73) (Figure 3a,c,e). To further evaluate whether these models perform well for new datasets (Figure 3b,d,f), 63,191 data points from the validation sets were used to input these models. The RMSEs for RFR were 35% and 85% lower than those for SVR in evaluations of the training set and validation set, respectively. The results show that the R^2^ values of these three models are 0.76–0.89, with the lowest RMSE value (4.7) in RFR for the validation sets. Based on the above evaluation, RFR is chosen as the best data correction model to be used for further applications.

### 3.3. Sensitivity Analysis of RFR

**Figure 4:**
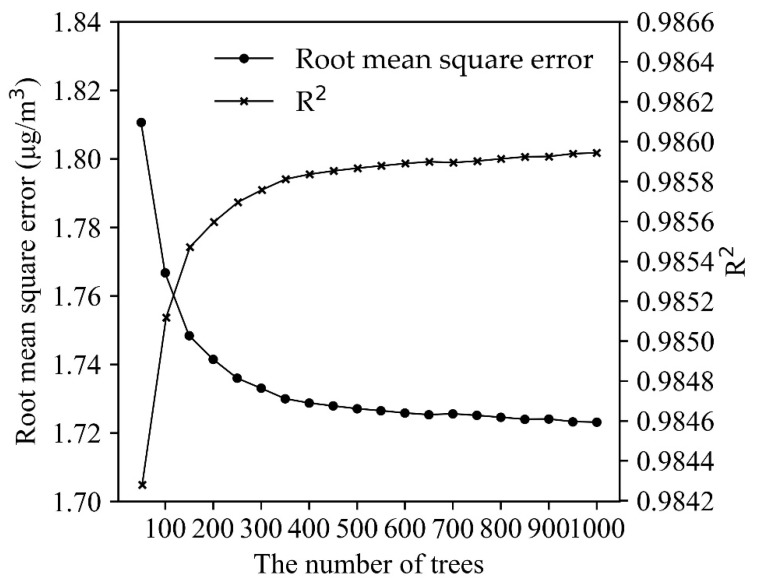
Model efficiency of the random forest regression model.

To optimize the computing efficiency of the RFR model, a sensitivity analysis of RFR was conducted with 50 to 1000 decision trees (with 50-tree increases in each simulation) to assess the changes in modeling efficiency. Figure 4 shows that RFR offers good model performance (RMSE = 1.81 and R^2^ = 0.9843) when there are only 50 trees. As the decision trees increase, the modeling efficiency is enhanced most significantly before the number of decision trees reaches 300. When the decision trees number is 300, the RMSE is 1.73, and the R^2^ is 0.9858. Afterward, the efficiency enhancement is not significantly altered by adding more decision trees, which takes more computing time. In the overall evaluations, RFR with 300 decision trees was chosen as the model with the best efficiency.

### 3.4. Comparison of the Model-Corrected PM_2.5_ and GRIMM-Calibrated PM_2.5_

#### 3.4.1. RFR with Whole-Day and Nighttime Patterns

A performance evaluation was further conducted for the RFR with a whole-day pattern using a whole-day dataset based on four seasons. Table 2 shows that the RMSE is the lowest in the summer model (mean: 5.4 μg/m^3^, ranging from 3.1 to 11.2 μg/m^3^), followed by the fall model (mean: 6.1 μg/m^3^, ranging from 3.7 to 10.0 μg/m^3^), while the RMSE values are slightly higher in the winter and spring models (mean: 6.8 and 7.3 μg/m^3^, respectively). For certain AS-LUNG-O sets at community locations, the r is as low as 0.33 between the model-corrected PM_2.5_ and GRIMM-calibrated PM_2.5_. This discrepancy is possibly caused by some nearby community sources that could not be detected by EPA monitoring stations. These community sources, such as traffic or restaurants, likely generate PM_2.5_ in the daytime. Therefore, AS-LUNG-O may have different PM_2.5_ trends from the nearby EPA station, leading to low correlations between these observations.

To focus on the systematic difference of the LCS and EPA observations, a data correction model was established for the nighttime dataset only. Since emissions from community sources resulting from human activity usually reached the lowest levels between 00:00 and 06:00, the data from this period were used to establish the model. Afterward, the established RFR with a nighttime pattern was used to correct the raw PM_2.5_ for all datasets (including both daytime and nighttime). This way, the readings of AS-LUNG-O were adjusted according to the systematic differences between the AS-LUNG-O and EPA instruments, while the extra PM_2.5_ increases due to community sources in the daytime could also be retained. Table 2 shows that the r-values of the model-corrected PM_2.5_ and GRIMM-calibrated PM_2.5_ were enhanced for the overall datasets, including both street-level and high-level AS-LUNG-O sets (including both daytime and nighttime). In the seasonal models, the r-values for RFR with a whole-day pattern were 0.83, 0.82, 0.85, and 0.90, which were improved to 0.92, 0.88, 0.88, and 0.94 for the RFR with a nighttime pattern for the spring, summer, fall, and winter models, respectively.

The model improvement is most obvious in spring for certain street-level AS-LUNG-O sets. Compared with the whole-day model with an r-value of 0.34, the r-value of the nighttime model is enhanced to 0.81 (Table 3). Nevertheless, the r-values of certain AS-LUNG-O sets were not improved with the nighttime models. It is possible that the trends of PM_2.5_ concentrations at these AS-LUNG-O locations were different from those in the EPA stations, regardless of whether it was during the day or night.

Furthermore, the results of the model evaluation were categorized as street-level and high-level (Table 3 and Table 4, respectively). For data correction at a high-level, the r-values between the model-corrected PM_2.5_ and GRIMM-calibrated PM_2.5_ are all above 0.68 in the whole-day models, while the nighttime models between them are all above 0.75. These results indicate that the PM_2.5_ levels at high-level AS-LUNG-O locations are moderately correlated with certain deviations from those of the EPA stations in a 3 km radius. This correlation was enhanced in the nighttime model. The minimum r-values of high-level AS-LUNG-O sets were improved from 0.68–0.83 to 0.75–0.86 (Table 4). This phenomenon was also observed in the PM_2.5_ correction of certain street-level AS-LUNG-O sets; the minimum r-values were improved from 0.33–0.79 to 0.57–0.89 (Table 3). However, the improvement of street-level correlations was not as good as those of high-level AS-LUNG-O sets. The PM_2.5_ levels sensed by street-level AS-LUNG-O locations are affected by local community sources, resulting in different PM_2.5_ patterns from those of the EPA stations. It is important to keep these local features while correcting LCS data with the systematic differences between LCS and research-grade instruments in the correction procedures. Thus, based on the evaluations of RFR with whole-day patterns and nighttime patterns, the latter were selected to correct the raw PM_2.5_ of AS-LUNG-Os.

#### 3.4.2. PM_2.5_ Corrections by RFR

**Figure 5:**
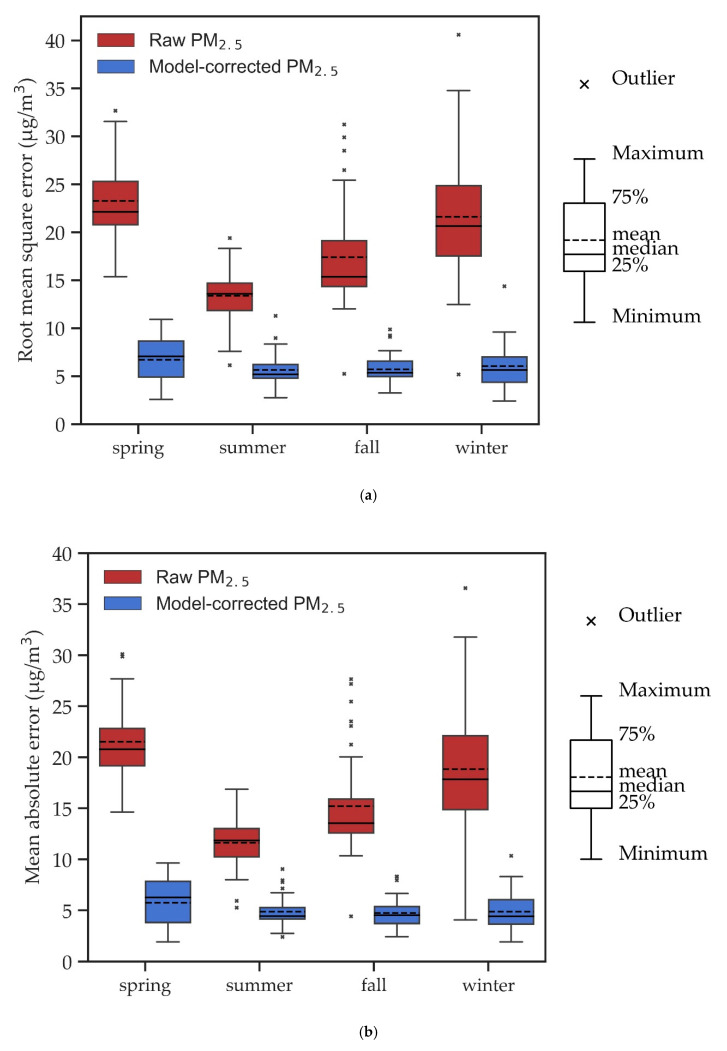
Correction results of RFR with a nighttime pattern for different seasons with (**a**) RMSE and (**b**) MAE as performance indicators.

Figure 5a shows the RMSE values between the GRIMM-calibrated PM_2.5_ and model-corrected PM_2.5_ using RFR with a nighttime pattern. The RMSEs were 2.6–10.9 (mean 6.7), 2.8–11.3 (mean 5.7), 3.2–9.9 (mean 5.7), and 2.4–14.4 (mean 6.1) μg/m^3^ for spring, summer, fall, and winter, respectively (Table 2). Before the model correction, the RMSE values were 15.4–32.7 (mean 23.3), 6.1–19.4 (mean 13.4), 5.3–31.2 (mean 17.4), and 5.2–40.6 (mean 21.6) μg/m^3^ for spring, summer, fall, and winter, respectively. Clearly, RFR can greatly reduce the RMSE values. The significant differences in RMSEs among different seasons almost disappeared after correction with RFR (the nighttime pattern).

In addition, the MAE is used to evaluate the performance of RFR compared to the models in the literature. Figure 5b shows MAE values between the model-corrected PM_2.5_ and GRIMM-calibrated PM_2.5_ with RFR (nighttime pattern). The MAEs were 1.9–9.6 (mean 5.7), 2.4–9.0 (mean 4.9), 2.4–8.3 (mean 4.8), and 1.9–10.3 (mean 4.9) μg/m^3^ for spring, summer, fall, and winter, respectively. Before the model correction, the MAE values were 14.6–30.1 (mean 21.5), 5.3–16.9 (mean 11.6), 4.4–27.6 (mean 15.2), and 4.1–36.6 (mean 18.8) μg/m^3^ for spring, summer, fall, and winter, respectively. In summary, after correction, the RMSE was improved from 18.4 ± 6.5 to 5.9 ± 2.0 μg/m^3^, and the MAE was improved from 16.2 ± 6.0 to 5.0 ± 1.8 μg/m^3^. This demonstrates that the RFR model greatly reduces MAEs, lowers the overestimation of AS-LUNG-O raw data, and improves the agreements between AS-LUNG-O and EPA PM_2.5_ levels.

**Figure 6:**
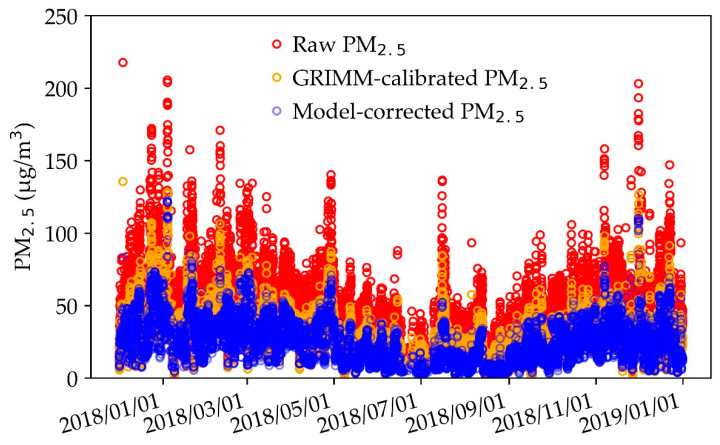
Time-series of raw PM_2.5_, model-corrected PM_2.5,_ and GRIMM-calibrated PM_2.5_.

Figure 6 shows a time series plot of the raw PM_2.5_ for AS-LUNG-O, the model-corrected PM_2.5_ with RFR (nighttime pattern), and the GRIMM-calibrated PM_2.5_ for the whole year of 2018. After learning from the PM_2.5_ observations of nearby EPA stations, the model-corrected PM_2.5_ levels were close to the GRIMM-calibrated PM_2.5_. In addition, after being corrected by machine learning techniques, the overestimation of the raw PM_2.5_ was greatly reduced. However, with nighttime models, the peak values of model-corrected PM_2.5_ were retained (as shown in the graph) to preserve the contributions of local community sources. After all, the purpose of community air quality monitoring is to assess the contributions of local sources; these important local features need to be preserved during the data correction processes.

## 4. Discussion

### 4.1. Comparison of in-Field PM_2.5_ Correction Models

Typically, environmental research groups apply monitoring instruments calibrated by the manufacturers. For LCS sensors, Rai et al. [48] proposed a two-stage calibration process with laboratory calibration performed by the manufacturers and calibration checks performed by the end-users. This process would be ideal if the manufacturers followed the suggestions. However, demanding manufacturers to calibrate LCSs may be unrealistic, since LCSs are made in larger quantities with much lower costs than more expensive instruments. Therefore, calibration needed to be carried out by the end-users as described in the introduction; most studies use laboratory evaluations before installing LCSs to establish correction equations. Moreover, only a few studies developed in-field data correction models for PM_2.5_ accuracy correction. Previously, we proposed a hybrid method combining laboratory evaluations and data science to ensure that LCS networks provide accurate PM data [20]. Statistical or machine learning methods were applied to adjust uncalibrated LCS devices with research-grade data within 3 km of the radius from either nearby EPA stations or calibrated seed LCS devices.

The focus of this work was to establish and evaluate three in-field data correction models for a PM_2.5_ LCS network—namely, the MLR, SVR, and RFR models. The AS-LUNG-O network, with individual correction equations for each LCS in the laboratory, offers a great opportunity to assess the performance of the data correction models established by machine learning techniques. Based on the results of the 10-fold cross-validation and holdout validation tests, there was only a little difference between the RMSEs of the 10-fold cross-validation and holdout validation tests for MLR, SVR, and RFR. Thus, to avoid overvaluing the performance of the models, we used the holdout validation to establish our models. Among them, RFR is the best model, with an RMSE of 1.73 and an R^2^ of 0.99 based on 63,190 raw hourly data of 39 AS-LUNG-O sets corrected with the PM_2.5_ levels of EPA stations located within a 3 km radius. In the validation set, RFR was not overfitted, and the data corrected with the RFR model agreed well with the EPA observations. Thus, RFR was chosen as the data correction model. This work demonstrated the applicability of RFR in correcting LCS networks. Previous studies used statistical and machine learning models with data from regulatory stations to correct side-by-side LCS sets on the same locations [15,30]. This work is the first to use data from monitoring stations to correct the data of the LCS network in other locations without monitoring stations. This work is unique in providing LCS data (GRIMM-calibrated PM_2.5_) based on laboratory evaluations for comparison in other locations.

Among the few in-field correction studies, one study applied the generalized additive model (GAM) to correct LCSs installed at three Taiwan EPA stations during December 2017 [15]. The RMSE values were reduced from 15.55–31.34 μg/m^3^ to 4.88–9.66 μg/m^3^ after correction. These results are similar to our data correction model results, showing RMSE values of 5.2–40.6 μg/m^3^ that were reduced to 4.3–9.6 μg/m^3^ (high-level PM_2.5_ corrected by RFR with nigh-time patterns in winter, see Table 4). Our correction model used similar input data. However, while an individual GAM model needed to be established for each LCS at least once every day [15], our model, with cumulative data of more than one year, was constructed as a one-time effort, which saved much computing time. Another study applied transfer learning to correct 10 months of PM_2.5_ data for LCSs located at seven public environmental monitoring stations in Beijing, China, and obtained MAEs of 7–12 μg/m^3^ [30]. The RFR model in our work obtained MAEs of 1.9–10.3 μg/m^3^ and performed at least comparable to or even better than those previous in-field data correction models for PM_2.5_. Furthermore, our methodology is also suitable to be applied to PM_2.5_ sensor networks in other countries.

One may concern that the upwind or downwind locations may also affect the relationship of AS-LUNG-O readings and EPA measurements. Since the prevailing wind in Taiwan changes with the season, the seasonal air flow variations were considered in the correction model by the variable of “season”. This variable “season” also considered all other seasonal factors.

In addition, in real-world applications, the computing efficiency of a model is a key issue to determine feasibility. The impressive computer efficiency of RFR is another advantage of the model, as it can handle huge datasets for the in-field correction of sensor networks. Based on the same training dataset, RFR needed only 0.23 minutes for training a model, while SVR needed approximately 8.16 minutes. In comparison, RFR was 35 times faster. With such fast computations, this method has great potential to be expanded to CAQN in Taiwan, which includes more than 4000 uncalibrated LCSs. Decreasing run time in the real-time corrections of CAQN can be carried out by increasing the computational capacity with a larger CPU and RAM. Moreover, parallel processing can be applied in model coding to speed up the correction task. Due to its fast computing efficiency, RFR is an excellent model for big data analytics for any data applications in sensor networks.

It should be noted that the RFR can only be applied for sensor networks with PM_2.5_ LCSs, which have good R^2^ with research-grade instruments. If the precision of PM_2.5_ LCS is not good, the RFR cannot adjust this inherent disadvantage. One previous study only enhanced the r-value between LCSs and research-grade instruments from 0.53 to 0.63 with RFR, since they used LCSs with R^2^ of 0.07 to 0.27 (compared to research-grade instruments) [49,50]. On the contrary, our present work used LCS devices with good R^2^ (>0.95) [10,20], so that our RFR model showed good performance in data correction for sensor networks. Thus, LCS with good precision is a prerequisite for a good data correction by RFR.

### 4.2. Limitations of This Work

There are some limitations to this work. RFR could greatly improve data accuracy, as shown by the reduction of RMSEs and MAEs. However, there is still an average bias of 4.8–5.7 μg/m^3^ for four seasons after correction. These deviations may come from the inherent differences in ambient (high-level) PM_2.5_ levels and street PM_2.5_ levels. The latter is affected by various community sources, while the former is measured by EPA monitoring stations purposely assessing well-mixed ambient PM_2.5_ levels free from any local emission interference. The AS-LUNG-O sets were located within 3 km radius of EPA stations. Theoretically, the PM_2.5_ levels were uniformly distributed within these distances. Nevertheless, there were usually multiple localized sources (home factories, restaurants, traffic, etc.) within 100–500 m in Taiwanese communities resulting in significant spatial variations. Therefore, the PM_2.5_ levels at AS-LUNG-O locations were different from those in EPA stations. This further demonstrates the necessity of establishing LCS networks to assess local ambient air.

On the other hand, the heights of AS-LUNG-O sets and EPA stations might be another variable for the correction model. Since there were only two types of height (ground level and high level) of AS-LUNG-O sets, we did not consider the altitude in the current correction model. This variable could be considered in the future.

Moreover, the performance of RFR may be improved by multiple year inputs, which may cover more environmental conditions for building the decision trees. This could be evaluated further after the accumulation of observations from the AS-LUNG-O network. Additionally, the co-localization of some AS-LUNG-O sets with EPA stations could be conducted in the future for comparison under the same environmental conditions.

## 5. Conclusions

Current data correction methods for PM_2.5_ sensor networks are mostly established in the laboratory and in the field before LCS installation. For citizen PM_2.5_ sensor networks without calibration, this work has developed an in-field data correction model with machine learning to adjust the accuracy deviations of the LCS network to enhance the data applicability of these networks. With the RFR model, the RMSEs and MAEs of the model-corrected PM_2.5_ and GRIMM-calibrated PM_2.5_ are greatly reduced. The contributions of local community sources to street-level PM_2.5_ concentrations are also preserved by RFR with a nighttime pattern (00:00 to 06:00). This work provides a feasible method for the in-field data correction of uncalibrated PM_2.5_ sensor networks with machine learning techniques. In addition, this work demonstrates the great potential of machine learning to enhance the agreement of LCSs and research-grade instruments, and thus, expands the applications of machine learning in the field of environmental monitoring.

Previously, we proposed a hybrid method combining laboratory evaluations and data science to ensure that LCS networks provide accurate PM data. Statistical or machine learning methods were applied to adjust uncalibrated LCS devices with data from nearby EPA stations or seed LCS devices that have been corrected by laboratory side-by-side comparisons and installed strategically in areas without EPA stations. The current work focuses on applying machine learning to correct the LCS network with PM_2.5_ from the Taiwan EPA. Under the trend of the smart city movement, increasingly more sensors will be installed in our living surroundings for air quality monitoring. Thus, accurate data are essential to avoid false impressions of better or worse air quality leading to ineffective air pollution control strategies. With the established in-field data correction models presented in this work and the calibrated seed LCS devices, accurate PM_2.5_ data from the sensor networks can be further applied to citizen science, public education, environmental research, and policymaking. Similar correction models can be established in other countries based on this example to greatly enhance the applicability and usefulness of PM_2.5_ sensor networks worldwide.

## Figures and Tables

**Table 1:** Seasonal mean with the standard deviation (SD), data range, and sample size (n) of the hourly raw PM_2.5_ of AS-LUNG-O sets and EPA PM_2.5_ (µg/m^3^).

	Raw PM_2.5_ of AS-LUNG		EPA PM_2.5_		*n*
	Mean (SD)	Range (Min, Max)	Mean (SD)	Range (Min, Max)	
Spring	48.0 (20.6)	(3.1, 295.9)	24.3 (13.6)	(2.0, 100.0)	19,924
Summer	28.4 (21.3)	(1.0, 249.8)	12.9 (13.0)	(2.0, 75.0)	37,638
Fall	36.8 (15.3)	(1.0, 223.6)	19.3 (8.1)	(2.0, 127.0)	43,624
Winter	51.5 (29.2)	(1.0, 309.8)	27.0 (18.2)	(2.0, 135.0)	25,195

**Table 2:** Performance evaluation of the random forest regression model (RFR) with whole-day and nighttime patterns in overall AS-LUNG-O sets (street-level and high-level).

Overall	Season	RMSE ^1^		Pearson Correlation		*n*
		Mean (SD ^2^)	Range (Min, Max)	Mean (SD)	Range (Min, Max)	
RFR with whole-day patterns	Spring	7.3 (2.6)	(4.1, 14.1)	0.83 (0.15)	(0.34, 0.96)	19,924
	Summer	5.4 (1.7)	(3.1, 11.2)	0.82 (0.11)	(0.33, 0.93)	37,638
	Fall	6.1 (1.6)	(3.7, 10.0)	0.85 (0.08)	(0.53, 0.94)	43,624
	Winter	6.8 (2.3)	(3.5, 12.9)	0.90 (0.04)	(0.79, 0.97)	25,195
RFR with nighttime patterns	Spring	6.7 (2.4)	(2.6, 10.9]	0.92 (0.05)	(0.80, 0.98)	19,924
	Summer	5.7 (1.7)	(2.8, 11.3)	0.88 (0.07)	(0.57, 0.95)	37,638
	Fall	5.7 (1.6)	(3.2, 9.9)	0.88 (0.08)	(0.68, 0.96)	43,624
	Winter	6.1 (2.3)	(2.4, 14.4)	0.94 (0.03)	(0.86, 0.98)	25,195

^1^ RMSE, root mean square error; ^2^ SD, standard deviation.

**Table 3:** Performance evaluation of the random forest regression model (RFR) with whole-day and nighttime patterns in street-level AS-LUNG-O sets.

Street-Level	Season	RMSE ^1^		Pearson Correlation		*n*
		Mean (SD ^2^)	Range (Min, Max)	Mean (SD)	Range (Min, Max)	
RFR with whole-day patterns	Spring	7.1 (2.7)	(4.1, 14.1)	0.84 (0.15)	(0.34, 0.96)	17,255
	Summer	5.4 (1.8)	(3.1, 11.2)	0.83 (0.11)	(0.33, 0.93)	30,710
	Fall	5.8 (1.5)	(3.7, 10.0)	0.85 (0.09)	(0.53, 0.94)	32,606
	Winter	6.5 (2.2)	(3.5, 12.9)	0.91 (0.04)	(0.79, 0.97)	19,448
RFR with nighttime patterns	Spring	6.5 (2.5)	(2.6, 10.9)	0.93 (0.04)	(0.81, 0.98)	17,255
	Summer	5.6 (1.8)	(2.8, 11.3)	0.89 (0.07)	(0.57, 0.95)	30,710
	Fall	5.7 (1.7)	(3.2, 9.9)	0.89 (0.08)	(0.68, 0.96)	32,606
	Winter	5.9 (2.4)	(2.4, 14.4)	0.94 (0.02)	(0.89, 0.98)	19,448

^1^ RMSE, root mean square error; ^2^ SD, standard deviation.

**Table 4:** Performance evaluation of the random forest regression model (RFR) with whole-day and nighttime patterns in high-level AS-LUNG-O sets.

High-Level	Season	RMSE ^1^		Pearson Correlation		*n*
		Mean (SD ^2^)	Range (Min, Max)	Mean (SD)	Range (Min, Max)	
RFR with whole-day patterns	Spring	8.8 (2.3)	(7.2, 10.4)	0.78 (0.15)	(0.68, 0.89)	2669
	Summer	5.7 (1.3)	(3.6, 7.6)	0.78 (0.07)	(0.70, 0.88)	6928
	Fall	7.3 (2.0)	(4.6, 9.9)	0.84 (0.07)	(0.75, 0.91)	11,018
	Winter	8.0 (2.9)	(4.6, 12.9)	0.88 (0.04)	(0.83, 0.94)	5747
RFR with nighttime patterns	Spring	8.2 (1.9)	(6.8, 9.5)	0.87 (0.09)	(0.80, 0.94)	2669
	Summer	5.8 (1.3)	(4.1, 7.9)	0.85 (0.06)	(0.76, 0.94)	6928
	Fall	6.2 (1.7)	(4.9, 9.3)	0.88 (0.09)	(0.75, 0.95)	11,018
	Winter	6.7 (2.2)	(4.3, 9.6)	0.92 (0.03)	(0.86, 0.94)	5747

^1^ RMSE, root mean square error; ^2^ SD, standard deviation.
